# An updated dose–response meta-analysis of coffee consumption and liver cancer risk

**DOI:** 10.1038/srep37488

**Published:** 2016-12-02

**Authors:** Chengbo Yu, Qing Cao, Ping Chen, Shigui Yang, Min Deng, Yugang Wang, Lanjuan Li

**Affiliations:** 1 State Key Laboratory for Diagnosis and Treatment of Infectious Diseases, The First Affiliated Hospital, School of Medicine, Zhejiang University, Collaborative Innovation Center for Diagnosis and Treatment of Infectious Diseases, Hangzhou, 310003, China; 2 Department of Gastroenterology, Shanghai Tongren Hospital, Affiliated to Shanghai Jiao Tong University School of Medicine, Shanghai, China

## Abstract

Prospective cohort studies of the relationship between coffee consumption and liver cancer risk have drawn different conclusions. Therefore, a dose-response meta-analysis of prospective cohort studies was performed to disentangle this causal relationship. Prospective cohort studies of the association between coffee consumption and liver cancer risk published prior to Jan 9, 2016 were identified by searching in the PubMed and EMBASE databases. Extracted data were analyzed using a random-effects model. Of the 2892 records identified using the search strategy, a total of twenty cohort studies from ten publications were included in the final meta-analysis. The pooled estimate of relative risk (RR) with 95% confidence interval (CI) for highest vs. non/occasional coffee drinkers was 0.55(0.44–0.67). No evidence of publication bias was observed (p for Egger’s test = 0.229). Sensitivity analysis indicated the results were robust. Dose-response analysis revealed a significant linear dose-response relationship between coffee consumption and liver cancer risk (p = 0.36). Subgroup analyses stratified by pre-specified variables (gender, geographic region, and adjusted factors) indicated similar results within individual subgroups. Our meta-analysis suggested that coffee consumption is inversely associated with liver cancer risk.

Primary liver cancer represents the second most common cancer-related cause of death and sixth most frequently diagnosed cancer worldwide^1^,^2^. Hepatocellular carcinoma (HCC) and intrahepatic cholangiocarcinoma (ICC) are the two dominant histologic types of primary liver cancer^3^. The aetiology of liver cancer is poorly studied. At present, well-documented unfavorable risk factors for liver cancer included chronic infection with hepatitis B virus (HBV) or C virus (HCV), excessive alcohol intake, and dietary aflatoxin^4^. The association between liver cancer and other risk factors, including obesity, diabetes mellitus, and smoking, is less clear^4^. Evidence from a recent meta-analysis indicated that increased intake of vegetables, but not fruits, appears to have a protective effect on HCC incidence^4^.

The effect of coffee intake on cancer risk has been a topic of considerable interest, because coffee is the second most popular beverage worldwide, ranking behind tea only. Coffee contains large amounts of compounds with antioxidant, anti-inflammatory, and anticarcinogenic properties such as caffeine, chlorogenic acid, caffeic acid, and polyphenols, as well as diterpenes and other antioxidants were found in that beverage^5^,^6^. Over the last two decades, numerous case-control and cohort studies have assessed the relationship between coffee intake and liver cancer risk; however, these studies have yielded inconsistent results. To disentangle this potentially confusing association, four meta-analyses of case-control and cohort studies had been performed^7^,^8^,^9^,^10^. All meta-analyses have suggested an inverse association between coffee intake and liver cancer risk. However, the tendency of retrospective case-control studies to be subject to recall and selection biases must be considered, as these biases may limit to the generalizability of the findings of these studies. In addition, the pattern of the dose-response curve between coffee exposure and liver cancer risk has not yet been described. Therefore, an updated dose-response meta-analysis of prospective cohort studies was performed.

## Methods

We report this meta-analysis according to the PRISMA (Preferred Reporting Items for Systematic Reviews and Meta-Analyses statement) guidelines^11^. There was no registered protocol. Ethical approval and patient consent were not necessary, because we conducted a meta-analysis of published studies.

### Inclusion Criteria

Studies were included if they fulfilled all the following criteria: (1) a prospective cohort study design was employed (prospective nested case-control studies were considered cohort studies); (2) the exposed population was patients with liver cancer; (3) the end point was incidence of or death from liver cancer; (4) the exposure of interest was coffee intake; and (5) adjusted RRs with corresponding 95 CIs were reported or other data provided were sufficient for their calculation. When more than one study studied the same population, the study reporting the most detailed information for both exposure and outcome was included in this meta-analysis.

### Data sources

A systematic literature search of the PubMed and EMBASE databases was performed to identify studies published prior to Jan 9, 2016. No restrictions on language or publication year were imposed. References of included studies, reviews, and meta-analyses were also reviewed to identify other potential studies, not identified using the initial search terms. Furthermore, studies citing the included studies in Google Scholar were manually reviewed.

### Search strategy

Two authors independently conducted the literature search. In our meta-analysis, we used the following key words or MeSH terms: (“coffee” OR “caffeine” OR “diet” OR “beverages” OR “lifestyle” OR “drinking”) AND (“hepatocellular carcinoma” OR “hepatic carcinoma” OR “liver cancer” OR “liver tumors” OR “liver neoplasms”) AND (“risk assessment” OR “risk” OR “risk factors”).

### Data extraction

The following data were independently extracted by two authors: first author last name, year of publication, cohort name, country, age of subjects at baseline, simple size, duration of follow-up (years), adjusted RRs with corresponding 95% CIs for each exposure level of coffee intake, and adjusted factors. Risk estimates that adjusted for the largest number of confounding factors were considered in the current meta-analysis. Any disagreements were resolved by discussion.

### Assessment the risk of bias

For observational studies, no well-established standard appraisal tools are currently available^12^. Therefore, the 9-star Newcastle-Ottawa Scale (NOS), which is recommended by the Cochrane Collaboration^13^, was adopted to assess the risk of bias (http://www.ohri.ca/programs/clinical_epidemiology/oxford.htm). Studies that scored seven stars or more were considered to be less prone to bias.

### Statistical Analysis

We derived a pooled estimate of adjusted RRs and 95% CIs using a random-effects model^14^, as the results provided by this model were more conservative. Heterogeneity across studies was tested using Cochran’s Q statistic and the proportion of total variation was estimated using I^2^ test^15^. We applied the following interpretation: I^2^ < 25% = low heterogeneity; 25% < I^2^ < 75% = moderate heterogeneity; and I^2^ > 75% = high heterogeneity. Publication bias was evaluated using funnel plots and Egger’s test^16^, where a p-value of the Egger’s test less than 0.05 suggests the presence of publication bias. In the sensitivity analysis, exclusion of single study at one time was assessed to investigate its influence of individual study.

For the primary analysis, we computed a pooled RR with 95% CI for highest vs. lowest intake of coffee. The lowest exposure category was defined as no consumption or occasional drinkers (0–1cup/day) of coffee. In subgroup analyses, we investigated the variations (gender, study region, adjusted factors) between studies. In addition, we performed a dose-response meta-analysis using the method described by Greenland and Orsini *et al*.^17^,^18^. Briefly, this method requires the exposure distribution of cases and person-years at risk, and RRs with 95% CIs for at least three quantitative exposure categories were provided in the original studies. For each study, the median or mean level of exposure within each category was assigned a corresponding RR; however, when unavailable, the midpoint of the upper and lower boundaries of each category was assigned as the value of exposure. When the highest category was open-ended, we assumed the length of the open-ended interval to be 1.5 times that of the closest category. A potential nonlinear or linear dose-response relationship between coffee consumption (cups/day) and liver cancer risk was modelled by using restricted cubic splines with 3 knots at fixed percentiles (25%, 50%, and 75%). A p-value for nonlinearity was calculated by testing the null hypothesis that the coefficient of the second spline was equal to 0^19^. All statistical analyses were performed using STATA/SE 12.0 software (StataCorp, College Station, TX, USA).

## Results

### Search results

The PRISMA statement flowchart shows the process of identification and study selection. (PRISMA_Flowchart_*SuppInfo.doc*). In the initial search, 2892 records were identified. We first excluded duplicates. A second assessment based on the reading of titles and abstracts was performed, and 2184 records were considered to be potentially eligible for inclusion. During the process of full-text evaluation, 4 records were excluded^20^,^21^,^22^,^23^. Finally, ten records were included in this meta-analysis^3^,^24^,^25^,^26^,^27^,^28^,^29^,^30^,^31^,^32^.

### Characteristics of the cohort studies

Table 1 shows the main characteristics of included studies. Ten records including twenty cohort studies, published from 2005 to 2015 were selected for inclusion. The studies were conducted in Japan, Singapore, the USA, Finland, Denmark, France, Germany, Greece, Italy, the Netherlands, Norway, Spain, Sweden, and the United Kingdom. Liver cancer cases in all included studies were identified by cancer or death registries. All included studies used cancer incidence as the end point with exception of the study conducted by Kurozawa *et al*.^25^, in which the endpoint was death from liver cancer. Of the studies, one reported data separately for HCC and ICC^3^; three assessed total primary liver cancer^26^,^27^,^30^, and the remaining included studies evaluated eHCC^24^,^25^,^28^,^29^,^31^,^32^.

### Risk of bias assessment

Table S1_SuppInfo.doc shows that all included studies received a score of seven stars or more; thus, included studies were judged to be at low risk of bias.

### Overall analysis

**Figure 1:**
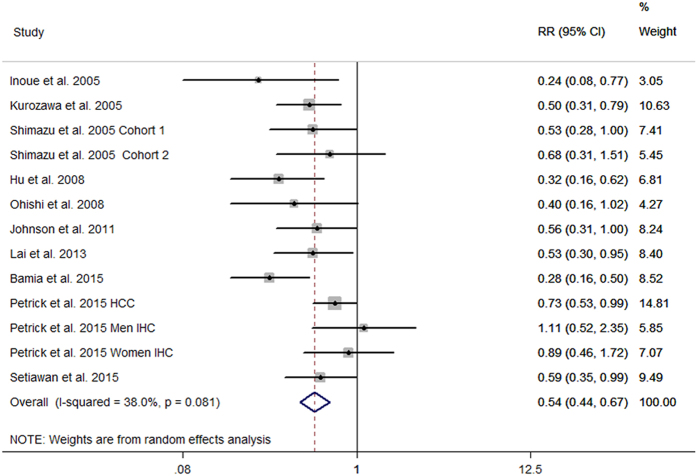
Forest plot for study-specific and pooled RRs and 95% CIs of liver cancer for highest versus lowest categories of coffee consumption. MHCC, hepatocellular carcinoma in men; WICC, intrahepatic cholangiocarcinoma in women; MICC, hepatocellular carcinoma in men.

Study-specific multivariable-adjusted RRs with 95% CIs for the highest versus lowest categories of coffee intake are shown Fig. 1. The pooled results showed an inverse relationship between coffee consumption and liver cancer risk. The summary RR and 95% CI were 0.55 (0.44–0.67), with moderate heterogeneity observed (I^2^ = 38.0%, P = 0.081).

### Sensitivity analysis

Sensitivity analysis indicated that the overall result was not substantially influenced by any single study, with RRs and 95 CIs ranging from 0.52 (0.41–0.65) to 0.59 (0.49–0.71), as shown in Table 2.

### Publication bias

**Figure 2:**
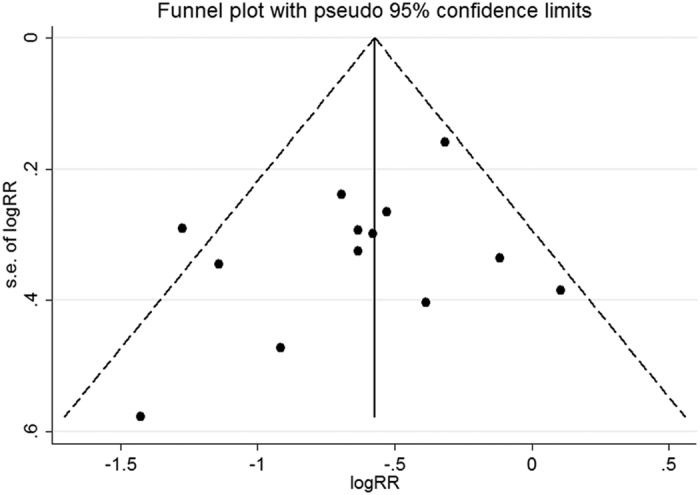
Funnel plot of liver cancer for highest versus lowest categories of coffee consumption. S.E. of logRR, Standard Error of logRR. The dashed lines represent the pseudo 95% CI. The sold line represents the effect estimate.

As shown in Fig. 2, the funnel plot did not show any asymmetry. Additionally, the result of Egger’s linear regression test suggested no evidence of publication bias (p = 0.229).

### Subgroup analysis

Table 3 shows the results of subgroup analyses stratified by gender, geographic region, and adjusted factors (history of liver diseases, history of diabetes, BMI, education, and tea consumption). No differences were identified in the association between coffee intake and liver cancer risk by gender (Male: RR = 0.58, 95% CI: 0.40–0.83, I^2^ = 59.5%; Female: RR = 0.57, 95% CI: 0.42–0.79, I^2^ = 0.0%). In the subgroup analysis of the effects of confounding factors on liver cancer risk, the results were not significantly modified by history of liver diseases, history of diabetes, BMI, education, or tea consumption (Table 3). When we stratified the analysis according to geographic region, a slight, moderate, and strong reduction in liver cancer risk was observed for studies conducted in North America (RR = 0.75, 95% CI: 0.59–0.95, I^2^ = 0.0%), Asia (RR = 0.50, 95% CI: 0.38–0.66, I^2^ = 0.0%), and Europe (RR = 0.37, 95% CI: 0.25–0.54, I^2^ = 22.6%), respectively.

### Dose-response analysis

**Figure 3:**
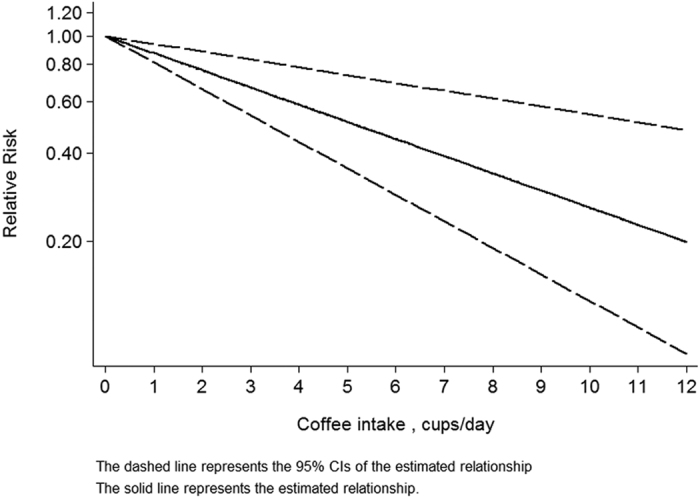
Dose-response relationships for the association between coffee consumption and liver cancer risk.

Eight publications providing necessary data were included in the dose-response analysis^3^,^24^,^25^,^26^,^27^,^29^,^31^,^32^. As shown in Fig. 3, a linear association between coffee consumption (cups/day) and risk of liver cancer was observed (P = 0.36). Compared with non-drinkers of coffee, the pooled RRs with 95% CIs were 0.87 (0.81–0.94), 0.76 (0.66–0.88), 0.67 (0.54–0.83), 0.58 (0.44–0.78), 0.51 (0.35–0.74), 0.44 (0.29–0.69) for consumption of 1 cup/day, 2 cups/day, 3 cups/day, 4 cups/day, 5 cups/day, and 6 cups/day, respectively.

## Discussion

### Main Findings

The current meta-analysis summarized evidence from twenty prospective cohort studies described in ten publications assessing the effect of coffee consumption on liver cancer risk. The results suggested that higher coffee intake was inversely associated with liver cancer risk. Analyses stratified by gender indicated that coffee intake was associated with a similar reduction in liver cancer risk in men and women. When the analysis was stratified by geographic region, a slight, moderate, and strong reduction in liver cancer risk was observed for observed in North American, Asian, and Europe an, respectively. Subgroup analyses performed to clarify the effects of confounding factors on liver cancer showed that our results were less susceptible to biases resulting from history of liver disease, history of diabetes, BMI, education, or tea consumption.

### Issues to further discuss and limitations

First, the types of coffee (caffeinated vs. decaffeinated coffee) associated with liver cancer risk were not differentiated. Only three studies provided separate data for caffeinated and decaffeinated coffee^3^,^31^,^32^. Findings from these three studies suggested a decreasing trend in HCC risk with increased caffeinated coffee consumption, however, no significant association was observed between decaffeinated coffee consumption and risk of liver cancer^3^,^31^,^32^. The significance of this discrepancy is unknown. Notably, limited numbers of subjects reporting decaffeinated coffee consumption were included in these studies. The null results were possibly due to limited statistical power. Moreover, only two studies quantified the effect of the amount of caffeine intake on liver cancer risk^29^,^32^. The Singapore Chinese Health Study reported that the RRs with 95% CIs for the 2nd (72–115 mg/day), 3rd (115–224 mg/day), and 4th (224–1,055 mg/day) quartile of caffeine intake were 1.09 (0.82–1.46), 0.82 (0.61–1.11), and 0.73 (0.54–1.00), respectively (p for trend = 0.02), when compared with the lowest quartile (0–722 mg/day)^29^. In the US Multiethnic Cohort, some interesting results were identified. Caffeine was associated with a decreased risk of HCC in models adjusting for age, sex, and ethnicity adjusted; however, when regular coffee was included as a confounding factor, the results were no longer statistically significant^32^. In consideration of the aforementioned findings, it’s possible that the inverse association between coffee consumption and liver cancer risk may not be largely a result of caffeine intake, and thus more studies are warranted to better explore the possibility of differential associations between caffeinated and decaffeinated coffee and liver cancer risk.

Second, the association between coffee consumption and liver cancer risk may vary by the method used for brewing coffee. Studies have shown that the presence of various compounds retained in coffee is dependent upon the preparation method of preparation. For example, compared with drip-filtered, percolated, or instant coffee, the amounts of cafestol and kahweol were found to be much higher in Scandinavian-styled boiled and Turkish coffee^33^,^34^. To our knowledge, the Alpha-Tocopherol, Beta-Carotene Cancer Prevention Study (ATBCCPS) has been the first prospective analysis of the relationship between liver cancer risk and coffee intake by coffee preparation method^30^. In the ATBCCPS of 20,737 men, boiling (about 21%) and filtering (about 71%) were the two most common methods of coffee preparation. Similar results were observed for consumption of boiled and filtered coffee.

Third, the dose effect of coffee intake duration or cumulative coffee intake prior to liver cancer diagnosis on liver cancer risk requires further elucidation. Some of included studies only reported data at baseline, which may not reflect changes in consumption habits following the date of study initiation. It is possible that non-coffee consuming individuals became coffee drinkers or individuals who consumed coffee at baseline may have stopped this consumption behavior after questionnaire administration. Thus, an over- or under-estimation of association between liver cancer risk and the true amount of coffee intake may have resulted.

Fourth, as a meta-analysis of published studies, potential publication biases may distort the results in either direction, however, no evidence for publication bias was observed.

Finally, the presence of unmeasured confounding inherited from original studies may be a matter of major concern. All included studies provided risk estimates derived from multivariable models (Table 1). However, only two studies provided risk estimates adjusted for age, sex, smoking, alcohol intake, history of liver disease, diabetes, and BMI. Thus, our findings may have been influenced if included studies failed to consider potential confounding factors.

### Comparison with previous meta-analyses and strengths

To elucidate this issue, five systematic reviews or meta-analyses have been published^7^,^8^,^9^,^10^,^35^. In a meta-analysis of four cohort and 5 case-control studies, Larsson *et al*. found that increased consumption of coffee may reduce the risk of liver cancer^7^. Three latter meta-analyses of cohort and case-control studies also found an inverse association between coffee consumption and liver cancer risk^8^,^9^,^10^. In 2016, Bravi *et al*. performed an updated meta-analysis of cohort studies and observed a 15% decreased risk in liver cancer for an increment of 1 cup of coffee per day^35^. In a model comparing regular, low, and high consumption with no or occasional coffee consumption, the pooled RRs with 95 CIs were 0.66 (0.55–0.78) for regular, 0.78 (0.66–0.91) for low, and 0.50 (0.43–0.58) for high coffee consumption, respectively.^35^ Compared with previous meta-analyses, our study had two major strengths. First, no retrospective case-control studies were included. This meta-analysis included twenty prospective cohort studies, which enhanced statistical power to detect significant associations. Additionally, potential biases related to recall, interviewers, and selection were greatly minimized. Second, an analysis of dose–response, which is considered to be an important criterion for the determination of causal relationships between exposure and disease in epidemiologic studies, was performed to quantify the relationship between coffee intake and liver cancer risk. We observed no evidence of statistically significant departure from linearity (P = 0.36). The pooled RRs with 95% CIs were 0.87 (0.81–0.94), 0.76 (0.66–0.88), 0.67 (0.54–0.83), 0.58 (0.44–0.78), 0.51 (0.35–0.74), and 0.44 (0.29–0.69) for 1 cup/day, 2 cups/day, 3 cups/day, 4 cups/day, 5 cups/day, and 6 cups/day, respectively.

## Conclusions

In conclusion, the results of our study provide evidence of a significant inverse linear dose-response association between coffee consumption and liver cancer risk.

## Additional Information

**How to cite this article**: Yu, C. *et al*. An updated dose–response meta-analysis of coffee consumption and liver cancer risk. *Sci. Rep.* **6**, 37488; doi: 10.1038/srep37488 (2016).

**Publisher's note:** Springer Nature remains neutral with regard to jurisdictional claims in published maps and institutional affiliations.

## Supplementary Material

Supplementary Information

## Figures and Tables

**Table 1:** Characteristics of included prospective studies of coffee consumption and risk of liver cancer.

Study	Cohort Name	Country	Age	No. of Cases	No. of Cohort size	Duration of follow-up (years)	Coffee consumption	Relative risk (95% CI)	Adjustment
Inoue *et al*.^24^	JPHC Study	Japan	40–69	334	90,452	10	≥5 cups/day vs. Almost never	0.24 (0.08–0.77)	Age, sex, study center, smoking, alcohol intake, vegetable consumption, and tea intake.
Kurozawa *et al*.^25^	JACC Study	Japan	40–79	258	83,966	11	≥1 cup/day vs. Non-drinkers	0.50 (0.31–0.79)	Age, sex, education, history of diabetes and liver diseases, smoking, and alcohol intake.
Shimazu *et al*.^26^	Cohort 1	Japan	>40	70	22,404	9	≥1 cup/day vs. Non-drinkers	0.53 (0.28–1.00)	Age, sex, history of liver disease, smoking, and alcohol intake
	Cohort 2	Japan	40–64	47	38,703	6	≥1 cup/day vs. Non-drinkers	0.68 (0.31–1.51)	Age, sex, history of liver disease, smoking, and alcohol intake
Hu *et al*.^27^	—	Finland	25–74	128	60,323	19.3	≥8 cups/day vs. 0–1 cup/day	0.32 (0.16–0.62)	Age, sex, study year, alcohol intake, smoking, education, diabetes, history of liver disease, and BMI
Ohishi *et al*.^28^	AHSI	Japan	NA	224	644	44	Daily vs. Non-drinkers	0.40 (0.16–1.02)	Age, sex, history of liver disease, alcohol intake, smoking, BMI, diabetes mellitus, and radiation dose to the liver.
Johnson *et al*.^29^	SCH study	Singapore	45–74	362	63,257	13	≥3 cups/day vs. Non-drinkers	0.56 (0.31–1.00)	Age, sex, dialect group, study year, BMI, education, alcohol intake, smoking, tea intake, and history of diabetes
Lai *et al*.^30^	ATBCP Study	Finland	50–69	194	27,037	18.2	≥4 cups/day vs. 0–1 cup/day	0.53 (0.30–0.95)	Age, BMI, education, marital status, history of diabetes, smoking, alcohol intake, tea intake, ATBC intervention arm, and serum cholesterol.
Bamia *et al*.^31^	EPIC	European	25–70	201	486,799	11	Q5 vs. Q1	0.28 (0.16–0.50)	Age, sex, diabetes, education, BMI, smoking, physical activity, alcohol intake, energy intake, and tea intake.
Petrick *et al*.^3^	LCPP	USA	25–70	1,120	1,212,893	10–22	>3 cups/day vs. Non-drinkers	MHCC:0.73 (0.53–0.99); MICC: 1.11 (0.52–2.35),WICC:0.89 (0.46–1.72)	Age, sex, race, cohort, BMI, smoking, and alcohol intake.
Setiawan *et al*.^32^	MEC	USA	45–75	451	162,022	18	≥4 cups/day vs. Non-drinkers	0.59 (0.35–0.99)	Age, sex, race/ethnicity, education, BMI, alcohol intake, smoking, and diabetes.

No., number; JACC Study: Japan Collaborative Cohort Study for Evaluation of Cancer Risk; JPHC Study: The Japan Public Health Center-based Prospective Study; AHSI, the Adult Health Study longitudinal cohort; SCH study, the Singapore Chinese Health Study; ATBCP Study:, the Alpha-Tocopherol, Beta-Carotene Cancer Prevention Study; EPIC, the European Prospective Investigation into Cancer and nutrition; LCPP, the Liver Cancer Pooling Project; MEC, the US Multiethnic Cohort; MHCC, hepatocellular carcinoma in men; WICC, intrahepatic cholangiocarcinoma in women; MICC, hepatocellular carcinoma in men.

**Table 2:** Results of sensitivity analysis.

Study excluded	Pooled RR with 95% CI	Heterogeneity	
		I^2^ (%)	P
Inoue *et al*.^24^	0.56(0.45–0.69)	35.7	0.105
Kurozawa *et al*.^25^	0.55(0.43–0.69)	42.3	0.060
Shimazu *et al*.^26^ Cohort 1	0.54(0.43–0.68)	43.0	0.056
Shimazu *et al*.^26^ Cohort 2	0.53(0.43–0.67)	42.5	0.059
Hu *et al*.^27^	0.57(0.46–0.70)	33.3	0.123
Ohishi *et al*.^28^	0.55(0.44–0.69)	41.5	0.065
Johnson *et al*.^29^	0.54(0.43–0.68)	43.1	0.055
Lai *et al*.^30^	0.54(0.43–0.69)	43.0	0.056
Bamia *et al*.^31^	0.59(0.49–0.71)	15.8	0.289
Petrick *et al*.^3^ HCC	0.52(0.41–0.65)	30.4	0.149
Petrick *et al*.^3^ Men IHC	0.52(0.43–0.64)	31.7	0.138
Petrick *et al*.^3^ Women IHC	0.52(0.42–0.65)	36.7	0.097
Setiawan *et al*.^32^	0.54(0.42–0.68)	43.0	0.056

**Table 3:** Summary risk estimates of the association between coffee consumption and risk of liver cancer.

Studies groups	References number	No. of cases/No. of total participants	RR (95% CI)	Heterogeneity	
				I^2^(%)	P
*All studies*	3,24–32	3,389/2,248,500	0.55(0.44–0.67)	38.0	0.081
*Study gender*					
Male	3,24–27,30	1,547/656,146	0.58(0.40–0.83)	59.5	0.022
Female	3,24–27	604/879,632	0.57(0.42–0.79)	0.0	0.588
*Study region*					
Asia	24–26,28,29	1,295/299,426	0.50(0.38–0.66)	0.0	0.763
Europe	27,30,31	523/574,159	0.37(0.25–0.54)	22.6	0.275
North America	3,32	1,571/1,374,915	0.75(0.59–0.95)	0.0	0.543
*Adjustment for tea*					
Yes	24,29,30,32	1,341/342,768	0.53(0.39–0.72)	0.0	0.557
no	3,25–28,31	2,041/1,905,732	0.55(0.41–0.74)	53.0	0.030
*Adjustment for history of liver diseases*					
Yes	25–28	727/204,060	0.48(0.36–0.63)	0.0	0.665
No	3,24, 29–32	2,662/2,044,440	0.58(0.43–0.78)	53.5	0.035
*Adjustment for history of diabetes*					
Yes	24,27–32	1,894/890,534	0.43(0.33–0.56)	9.9	0.353
No	3,25,26	1,495/1,357,966	0.69(0.56–0.84)	0.0	0.453
*Adjustment for BMI*					
Yes	3,27–32	2,680/2,224,975	0.56(0.42–0.74)	51.5	0.036
No	24–26	709/235,525	0.50(0.36–0.70)	0.0	0.527
*Adjustment for education*					
Yes	24,27,29–32	1,670/889,899	0.43(0.32–0.58)	24.6	0.249
No	3,25,26,28	1,719/1,358,601	0.67(0.54–0.82)	0.0	0.430

No., Number.
