# Supplementary material for: An updated dose–response meta-analysis of coffee consumption and liver cancer risk
Source: Sci Rep. 2016 Dec 2;6:37488. doi: 10.1038/srep37488 (PMC5133591; doi:10.1038/srep37488)
Supplement: Supplementary Information [file srep37488-s1.pdf]

An updated dose–response meta-analysis of coffee consumption and liver cancer risk

Chengbo Yu[1]#, MD, Qing Cao[1]# MD, Ping Chen[1] MD, Shigui Yang[1] PhD, Min Deng[1] PhD, Yugang Wang[2*] Lanjuan Li[1*] MD

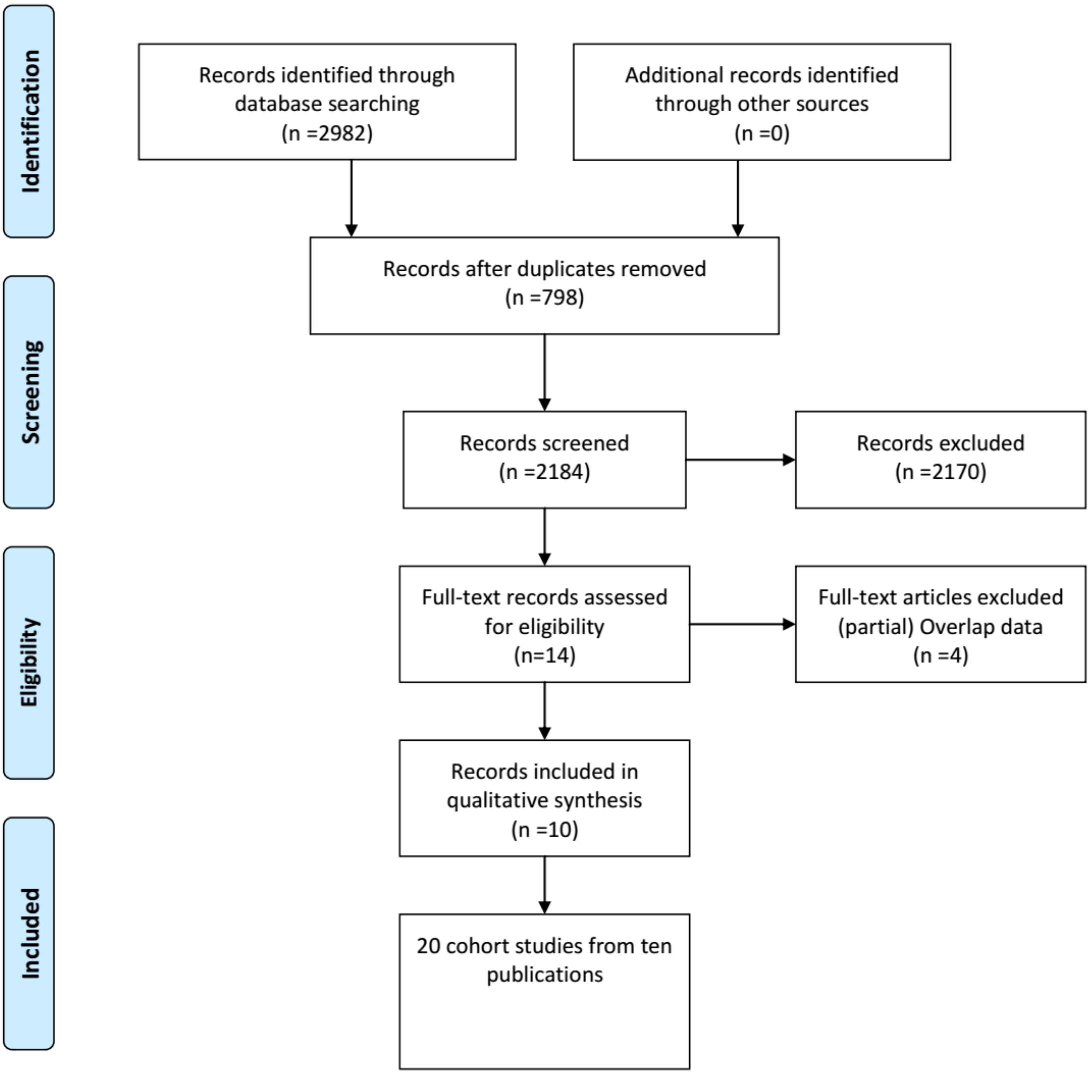

PRISMA_Flowchart_SuppInfo.doc Flow diagram of study selection.

Table S1_SuppInfo.doc Assessment of bias risk of included studies.

**Study**		**Selection**			**Comparability**[1]		**Outcome**		
	**Representativeness of the exposed cohort**	**Selection of the unexposed cohort**	**Ascertainment of exposure**	**Outcome of interest not present at start of study**	**Control for important factor or additional factor**	**Assessment of outcome**	**Follow-up long enough for outcomes to occur**[2]	**Adequacy of follow-up of cohorts**[3]	**Total quality scores**
Inoue et al. 2005	☆	☆	☆	☆	☆	☆	☆	☆	8
Kurozawa et al. 2005	☆	☆	☆	☆	☆	☆	☆	☆	8
Shimazu et al. 2005	☆	☆	☆	☆	☆	☆	☆	☆	8
Hu et al. 2008	☆	☆	☆	☆	☆☆	☆	☆	☆	9
Ohishi et al. 2008	---	☆	☆	☆	☆☆	☆	☆	☆	8
Johnson et al. 2011	☆	☆	☆	☆	☆	☆	☆	☆	8
Lai et al. 2013	--	☆	☆	☆	☆	☆	☆	☆	7
Bamia et al. 2015	☆	☆	☆	☆	☆	☆	☆	☆	8
Petrick et al. 2015	☆	☆	☆	☆	☆	☆	☆	☆	8
Setiawan et al.2015	☆	☆	☆	☆	☆	☆	☆	☆	8

[1] A maximum of two stars can be assigned for comparability. In current studies, two stars could be assigned, if the included studies provided risk estimates adjusted for age, sex, smoking, alcohol intake, history of liver diseases, diabetes, and BMI. Otherwise, no more than one star could be assigned.

[2] One star was assigned to a cohort study with five follow-up years or more.

[3] A cohort study with a follow-up rate > 75% was awarded one star.
